# Bioactive peptides from *Chlamydomonas reinhardtii* protein hydrolysate: Identification, antimicrobial activity, and mechanism of action

**DOI:** 10.1016/j.fochx.2025.103140

**Published:** 2025-10-09

**Authors:** Keying Su, Lecheng Wu, Yating Lin, Qian Li, Hua Liu, Xuewu Zhang, Lai-Hoong Cheng

**Affiliations:** a College of Engineering, Guangzhou College of Technology and Business, 510850, Guangdong, China; b Food Technology Division, School of Industrial Technology, Universiti Sains Malaysia, 11800 USM, Penang, Malaysia; c College of Food Science and Engineering, South China University of Technology, 510641 SCUT, Guangdong, China

**Keywords:** *Chlamydomonas reinhardtii*, Antimicrobial peptides, *Salmonella*, Mechanism, Molecular docking

## Abstract

Algal-derived peptides are gaining attention as potential natural bio-preservatives. This study aimed to identify antimicrobial peptides from *Chlamydomonas reinhardtii* protein hydrolysates against *Salmonella*. Protein hydrolysates were prepared, fractionated, and screened for antimicrobial activity, and the optimal fraction TC3–10 was selected for mechanisms elucidation, peptides identification and in silico analysis. Results showed that TC3–10 displayed the continuously strong inhibitory effect at the concentraction at 0.25–1 mg/mL of 40.24 ± 6.94 %-46.36 ± 3.86 %, primarily through membrane disruption, leakage of intracellular components, and Na^+^/K^+^-ATPase activity reduced. After in silico screening, peptide EWRPF showed high affinity potential against LuxS of −132.4 with six hydrogen bonds and two π-stacking, and GyraseA C-terminal domain of −133.6 with two hydrogen bonds and three π-stacking. This work provided the first evidence of antimicrobial peptides from *C. reinhardtii* hydrolysates, combining experimental validation with in silico prediction to highlight their potential as novel bio-preservatives.

## Highlights

• Strong inhibition of *Salmonella* spp. by *C. reinhardtii* protein hydrolysate (TC3–10).
• Dual antibacterial mechanism: membrane disruption and enzyme activity reduction.
• Peptide EWRPF with high binding affinity to *Salmonella* enzymes (molecular docking).
• *C. reinhardtii*-derived peptides as promising natural food preservatives.

## 1 Introduction

The extensive use of synthetic chemical preservatives and antibiotics in food and aquaculture systems has raised serious concerns due to antimicrobial resistance (AMR), residual toxicity, and environmental impacts. The World Health Organization (WHO) recognizes AMR as one of the top ten global public health threats, attributing an estimated 700,000 deaths annually to drug - resistant infections, with projections reaching 10 million deaths by 2050 (O'Neill, 2016). Although chemical preservatives such as benzoates, sorbates, and nitrites are widely used to inhibit microbial growth, they have been associated to adverse health effects, including allergenicity, carcinogenic by-products, and gut microbiota disruption (Wei & Zhang, 2022). Given rising concerns over resistance and food safety, natural and safer alternatives are urgently needed. Antimicrobial peptides (AMPs), small gene-encoded molecules present in the innate immunity of diverse organisms, represent promising candidates (Hancock & Sahl, 2006). They exhibit broad-spectrum activity against bacteria, fungi, viruses, and parasites, with low resistance potential and environmental compatibility. Their distinctive mechanisms, including membrane disruption and intracellular interference, provide clear advantages over conventional antibiotics.

Microalgae represent efficient and sustainable reservoirs of antimicrobial peptides, combining high protein yields with cost-effective large-scale cultivation and minimal resource requirements (Amiri et al., 2025; Ayswaria et al., 2023). *Chlamydomonas reinhardtii* presents distinct advantages as a source of antimicrobial peptides. Unlike many other microalgae, it is a well-studied model organism with a sequenced genome (Merchant et al., 2007), advanced genetic tools, and reliable cultivation systems. Its rapid growth, high protein content, and scalability under cost-effective and eco-friendly conditions further enhance its potential. Compared to other algae with lower protein yields and less standardized cultivation, *C. reinhardtii* offers a unique platform for discovering and producing bioactive peptides, supporting its application in sustainable antimicrobial strategies. Several microalgae, including *C. reinhardtii*, *Spirulina platensis*, and *Tetraselmis suecica*, have been identified as underexplored AMP reservoirs. Peptides from *S. platensis* hydrolysates showed strong activity against *B. subtilis* and *E. coli* (Lastra et al., 2025), while *C. reinhardtii* extracts inhibited *S. aureus* and *P. aeruginosa* (Jayshree et al., 2016). Transgenic *C. reinhardtii* strains expressing exogenous AMPs also displayed potent antimicrobial effects (Dubey et al., 2024).. However, studies on native protein hydrolysates remain scarce, and the intrinsic AMPs of *C. reinhardtii*, along with their structures and mechanisms, are still poorly understood.

The antimicrobial mechanisms of AMPs are broadly classified into membrane targeting and intracellular interference. Membrane-targeting AMPs disrupt microbial membranes through pore formation or depolarization, as shown by cecropin-like peptides from *Spirulina platensis* (Sun et al., 2016) and ALFPm3 expressed in *C. reinhardtii*, which binds lipopolysaccharides and destabilizes bacterial membranes (Li et al., 2021). In contrast, intracellular-interference AMPs penetrate cells and inhibit essential processes by binding nucleic acids or enzymes, such as DNA gyrase inhibition by *Chondrus crispus* peptides (Habibie et al., 2023) and DNA-binding activity of *Chlorella*-derived peptides (Sneideris et al., 2023), similar to the mechanism of Buforin II.

In recent years, in silico approaches have transformed antimicrobial peptide (AMP) research. Bioinformatics tools, such as PeptideRanker and the Antimicrobial Peptide Calculator and Predictor enable the prediction of bioactive peptides and their potential antimicrobial activity based on amino acid sequences (Tikhonov et al., 2025). Molecular docking is a common computational approach that predicts peptide–protein interactions, providing insights into binding affinity and stability. Zhang et al. (2024) combined de novo transcriptome sequencing and molecular docking to identify AMPs from *Aureococcus anophagefferens* active against *Escherichia coli*, where selected peptides exhibited strong binding to bacterial membranes and correlated with a low minimum inhibitory concentration (MIC) of 31.25 μg/mL*.* Similarly, peptides extracted from the marine macroalgae *Chondrus crispus* were screened for amphipathicity and subsequently docked against bacterial enzymes such as dihydrofolate reductase (DHFR) and DNA gyrase, confirming their potential antimicrobial roles (Habibie et al., 2023). Collectively, these in silico strategies not only reduce time and experiment costs but also provide a rational framework for the design and optimization of AMPs with improved activity and specificity.

Despite the growing interest in microalgal antimicrobial peptides (AMPs), research on those derived from *Chlamydomonas reinhardtii* remains limited and fragmented. Previous studies have only suggested the presence of potentially bioactive peptides in *C. reinhardtii* protein hydrolysates (Jayshree et al., 2016; Mao et al., 2025), yet these peptides were neither comprehensively identified nor mechanistically characterized, leaving a critical knowledge gap. Importantly, *C. reinhardtii* offers unique advantages as a source of functional proteins and peptides, including ease of cultivation, high biomass productivity, environmental sustainability, and rich protein content, all of which highlight its strong potential for large-scale development. Considering the urgent need for natural and sustainable bio-preservatives to enhance food safety and to provide alternatives to conventional antibiotics, it is both timely and necessary to explore *C. reinhardtii* as a novel reservoir of antimicrobial peptides.

This study addresses the gap by systematically investigating the antimicrobial peptides derived from *C. reinhardtii* protein hydrolysates. Specifically, the study innovatively combines traditional experiment method and in silico analysis to srceen antimicrobial peptides from *C. reinhardtii,* meanwhile elucidates their mechanisms of action. By integrating biochemical and computational approaches, this work provides the first in-depth insight into the antimicrobial potential of *C. reinhardtii*-derived peptides, highlighting their promise as innovative candidates for food preservation and broader biotechnological applications.

## 2 Materials and methods

### 2.1 Materials

The freeze-dried *C. reinhardtii* powder was purchased from Touyun Biotech in Shanxi, China. The *Salmonella* (CICC 10437) strain was procured from Biobw Biotechnology Co., Ltd. (Beijing, China). PI/RNase staining solution, SYBR Green I dye were obtained from Yining Biotechnology Co., Ltd.(Guangzhou, China). LST broth(Macklin), Trypsin(CAS 9002-07-7, USP grade, 130 u/mg), Chymotrypsin (CAS 9004-07-3, USP grade, 1500 u/mg), Bradford Protein Assay Kit(Beyotime, P0006), AKP assay kit (Beyotime, P0321S) and Na^+^/K^+^-ATPase assay kit (Beyotime, P0322S) were used, and Centrifugation tube(Millipore; 15 mL; 3 kDa and 10 kDa;), Dialysis membrane (Jiele Pu, 500 Da) were employed in this study.

### 2.2 Extraction of *Chlamydomonas reinhardtii* protein

*C. reinhardtii* protein was extracted following combined freeze–thaw and high-pressure treatments. Briefly, algal powder was suspended in double-distilled water, soaked for 2 h, and subjected to three freeze–thaw cycles (−80 °C/37 °C) to disrupt the cell wall (Wu et al., 2017). The suspension was then treated with high-pressure processing (300 MPa, 20 min, two cycles, 25 °C) to further enhance protein release (Braspaiboon & Laokuldilok, 2025), followed by centrifugation at 8900*g* for 10 min. Proteins in the supernatant were precipitated with ammonium sulfate (100 % saturation, 4 °C, overnight), collected by centrifugation, dissolved in water, and dialyzed using a 500 Da MWCO membrane to remove small molecules. Finally, the crude protein solution was lyophilized for 72 h to obtain *C. reinhardtii* protein samples.

### 2.3 Preparation and fractionation of *Chlamydomonas reinhardtii* protein hydrolysates

Lyophilized *C. reinhardtii* protein was dissolved in double-distilled water to 3 % (*w*/*v*). Hydrolysis was carried out using trypsin, chymotrypsin, or their 1:1 mixture under pH 7.8 and 40 °C for 240 min. Enzyme-to-substrate ratios were 6 % (*w*/w) for trypsin, 3 % (w/w) for chymotrypsin, and 6 % (w/w, 1:1) for the mixture. After hydrolysis, samples were centrifuged at 8900*g* for 10 min, and the supernatants fractionated by ultrafiltration. Hydrolysates were sequentially filtered through 3 kDa and 10 kDa membranes (5000*g*, 30 and 20 min, respectively) to yield fractions <3 kDa, 3–10 kDa, and > 10 kDa. Each hydrolysate was collected separately and lyophilized for 72 h, resulting in nine peptide fractions (coded as shown in Table 1). (See Table 1.)

**Table 1:** Abbreviations used for peptide fractions derived from *Chlamydomonas reinhardtii* protein hydrolysates after enzymatic digestion and ultrafiltration.

Molecular weight range	Enzymatic hydrolysis treatment by		
	Trypsin	Chymotrypsin	“Trypsin- Chymotrypsin” Combination
<3 kDa	T3	C3	TC3
3–10 kDa	T3–10	C3–10	TC3–10
>10 kDa	T10	C10	TC10

Note: T, C and TC represent trypsin, chymotrypsin and their combination, respectively. The number indicates peptide molecular weight range.

**Table 1:** Abbreviations used for peptide fractions derived from *Chlamydomonas reinhardtii* protein hydrolysates after enzymatic digestion and ultrafiltration.

Molecular weight range	Enzymatic hydrolysis treatment by		
	Trypsin	Chymotrypsin	“Trypsin- Chymotrypsin”
			Combination
<3 kDa	T3	C3	TC3
3–10 kDa	T3–10	C3–10	TC3–10
>10 kDa	T10	C10	TC10

Note: T, C and TC represent trypsin, chymotrypsin and their combination, respectively. The number indicate peptide molecular weight range.

### 2.4 In vitro antimicrobial activity

Standard *Salmonella* spp. strain (CICC 10437) was used. A single colony was cultured in 5 mL LST broth at 37 °C for 12 h, diluted 1:100 in fresh broth, and incubated to mid-log phase (10^7^–10^8^ CFU/mL). The suspension was adjusted to OD₆₀₀ = 0.1. *C. reinhardtii* peptide fractions were dissolved in double-distilled water to 0.25–2 mg/mL. For antimicrobial assays, 96-well plates were prepared with 100 μL peptide solution and 100 μL bacterial suspension per well, followed by incubation at 37 °C for 24 h. Controls contained only bacteria and broth. Growth inhibition was determined at OD₆₀₀ using a microplate reader, and fractions with higher activity were selected for mechanism studies.

(1) $$\text{Inhibitory activity}\;\left(\%\right)=\frac{A_{1}-A_{2}}{A_{1}}\times 100$$

where A_1_ is OD_600_ of control group, A_2_ is OD_600_ of test group.

### 2.5 Antimicrobial mechanism

#### 2.5.1 Samples preparation

For all antimicrobial mechanism assay, the *Salmonella* spp. cultured to the logarithmic growth phase were washed three times with phosphate-buffered saline (PBS) and resuspended to an adjusted concentration of 10^7^ CFU/mL. Peptide solutions were added to the bacterial suspensions to achieve final concentrations of 62.5 μg/mL, 31.25 μg/mL, and 15.625 μg/mL, respectively. Three replicates were prepared for each group, with PBS used as the blank control.

#### 2.5.2 Propidium iodide (PI) staining analysis

Samples were incubated at 28 °C with shaking for 4 h, and 1 mL aliquots were collected, washed three times with double-distilled water, and adjusted to 10^6^–10^7^ CFU/mL. PI staining was performed following Dong et al. (2022) with minor modifications. Briefly, bacterial cells were centrifuged at 7120*g* for 10 min, resuspended in 0.5 mL PI/RNase solution (10 μg/mL), and incubated in the dark for 15 min. After two washes with double-distilled water, the cells were resuspended and analyzed by fluorescence spectroscopy, Flow cytometric analysis was performed using a Beckman Coulter CytoFLEX flow cytometer equipped with a 488 nm argon-ion laser for excitation. PI fluorescence was detected in the FL2 channel using a 585/42 band-pass filter. FSC and SSC were simultaneously collected to distinguish intact cells from debris. A total of at least 10,000 events were recorded per sample.

#### 2.5.3 Relative conductivity measurement

Followed by treated with process in 2.4.1, samples were incubated in a shaking incubator at 28 °C for 12 h, and the relative conductivity of the solution was measured every 2 h with a conductivity meter (EC112, Thermofisher).

#### 2.5.4 Extracellular protein content

The samples outlined in section 2.4.1 were subsequently subjected to an 8-h incubation in a shaking incubator maintained at 28 °C. The protein content was subsequently determined using the Coomassie Brilliant Blue G-250 assay.

#### 2.5.5 Extracellular nucleic acid content

Following the procedure described in section 2.4.1, samples were incubated at 28 °C with shaking for 8 h. Nucleic acid content was quantified using a fluorescence system (QuantStudio™ 5, Thermo Fisher). Each 20 μL reaction contained 10 μL PBS buffer, 1 μL SYBR Green I dye, 1 μL sample, and 8 μL deionized water. After gentle mixing, reactions were loaded into fluorescence tubes and measured at 497 nm (excitation) and 520 nm (emission). Nucleic acid concentrations were calculated using a standard curve of nucleic acid standards. All assays were performed in triplicate, with PBS as the negative control.

#### 2.5.6 Alkaline phosphatase (AKP) activity

Incubation of the samples (as described in 2.4.1) was carried out in a shaking incubator at 28 °C for a duration of 12 h. Samples were taken and centrifuged at 5340*g* for 5 min to remove the supernatant and collect the pellet. The pellet was washed three times with PBS by centrifugation. Samples were subjected to cell disruption using an ultrasonic disruptor (JY96-IIN, SCIENTZ) in an ice-water bath. The parameters were as follows: power of 100 W, ultrasonic duration of 5 s each time, interval of 10 s, and total disruption time of 2 min. The activity of alkaline phosphatase (AKP) in the samples were detected using an AKP assay kit (Beyotime, P0321S).

#### 2.5.7 Na^+^-K^+^ATPae activity

The samples specified in 2.4.1 were then placed in a shaking incubator at 28 °C and incubated for 8 h. Samples were taken and the cells were disrupted by ultrasonication in ice-water bath in 100 W for 2 min, with ultrasonication for 5 s followed by a 10-s interval. The Na^+^/K^+^-ATPase in the disrupted bacterial suspensions were processed using a Na^+^/K^+^-ATPase assay kit (Beytime, P0322S).

### 2.6 Identification of peptides

Peptide fractions with the highest antimicrobial activity were dissolved in 20 μL of 0.1 % formic acid, vortexed, and centrifuged at 17,000*g* for 20 min at 4 °C. A 3 μL aliquot of the supernatant was analyzed by RPLC-MS using a mobile phase of 0.1 % formic acid (A) and 0.1 % formic acid in acetonitrile (B). The gradient program was: 95 % A (0–8 min), 90 % A (8–33 min), 85 % A (33–43 min), 72 % A (43–50 min), 60 % A (50–60 min), and 5 % A (60–65 min). MS parameters were: MS1—resolution 70,000, AGC target 3e6, maximum IT 60 ms, scan range 300–1400 *m*/*z*; MS2—resolution 17,500, AGC target 5e4, maximum IT 80 ms, TopN 20, NCE 27.

### 2.7 PEAKS studio analysis

Peptide sequences identification was achieved by automatic de novo sequencing function in PEAKS Studio according to the MS results. The parameters were set as 15 ppm for Precursor Mass Tolerance, 0.05 Da for Fragment Mass Tolerance, Oxidation(M) and Acetylation(N-term) for Dynamic Modifications.

### 2.8 In silico analysis of identified peptides sequences

Peptide sequences with Average Local Confidence (ALC) >90 % from PEAKS Studio were selected for in silico analysis. Bioactivity potential was first evaluated using PeptideRanker (http://distilldeep.ucd.ie/PeptideRanker/), with scores >0.8 considered bioactive. Antimicrobial properties were further predicted with APD3 (https://aps.unmc.edu/) based on charge, GRAVY, and Boman indices. Peptide toxicity was assessed using ToxinPred (http://crdd.osdd.net/raghava/toxinpred/), which applies a virtual scanning method (VSM) trained on Swiss-Prot data and a support vector machine (SVM) threshold of 0.0.

### 2.9 Molecular docking analysis

DoGSiteScorer module on the Protein Plus platform (https://proteins.plus/) was used to predicted protein pockets. The protein structures were further processed in Discovery Studio 2024 to remove water molecules and ligands, and add hydrogen atoms. Molecular docking were subsequently performed against two key proteins: LuxS from *salmonella typhi*(PDB ID: 5v2w) (Kint et al., 2009) and GyraseA C-Terminal Domain from *Salmonella typhi*(PDB ID: 5ztj) with the MDockPeP online platform (https://zougrouptoolkit.missouri.edu/MDockPeP/). Protein 5v2w represents an enzyme conserved across Gram-positive and Gram-negative bacteria that catalyzes the conversion of S-ribosylhomocysteine to DPD, the precursor of autoinducer-2, a key quorum-sensing molecule to adjust virulence secretion (Rezzonico & Duffy, 2008; Schauder et al., 2001). Protein 5ztj is type II topoisomerase that essential for DNA replication and transcription, which is indispensable for bacterial survival(Zeiske et al., 2018). The resulting docking poses were imported into Protein Plus, where interaction analysis was conducted using PoseView for 2D molecular interaction diagrams visualization.

### 2.10 Statistical analysis

All experiments were performed in triplicate with three independent repetitions. Data were analyzed using Microsoft Excel 2019, and statistical comparisons were conducted by one-way ANOVA followed by Tukey's test in SPSS 27. Differences were considered significant at *p* < 0.05. Results are presented as mean ± standard deviation (SD).

## 3 Results

### 3.1 Protein content and degree of hydrolysis

**Fig. 1:**
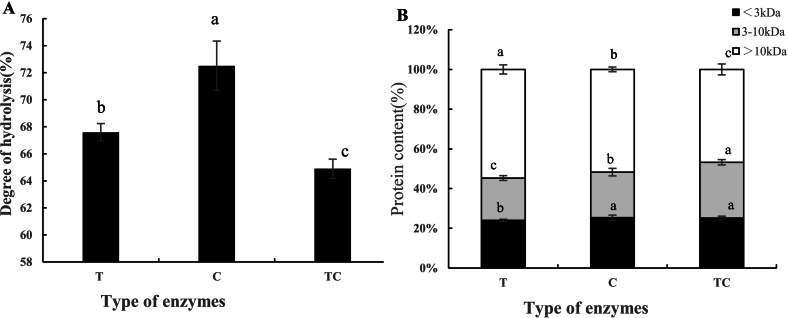
Degree of hydrolysis of *Chlamydomonas reinhardtii* protein (A) and protein distribution of peptide fractions (B) obtained by different enzymatic treatments (trypsin [T], chymotrypsin [C], and trypsin–chymotrypsin combination [TC]). Peptide fractions were classified by molecular weight into <3 kDa, 3–10 kDa, and > 10 kDa. Different lowercase letters indicate statistically significant differences between enzyme treatments (*p* < 0.05).

Fig. 1(a) shows the degree of hydrolysis (DH) of *C. reinhardtii* protein treated with three different enzymatic systems: trypsin (T), chymotrypsin (C), and a trypsin–chymotrypsin composite system (TC). Among these, chymotrypsin resulted in the highest DH at 72.51 %, significantly greater than trypsin (67.59 %) and the TC combination (64.89 %) (*p* < 0.05). This suggests that a more aggressive proteolytic action on *C. reinhardtii* protein is exerted by chymotrypsin, possibly due to its preference for cleaving at the hydrophobic residues (Phe, Tyr, Trp), which are known to be abundant in algal proteins (Bleakley & Hayes, 2017). Interestingly, the TC group showed the lowest DH, which may reflect interference between the two enzymes' cleavage preferences, leading to suboptimal access to peptide bonds. However, different restriction sites of two enzymes may exhibit more possibility to produce peptide with bioactivity.

Fig. 1(b) shows the protein content of different peptide fractions classified by different molecular weight range (<3 kDa, 3–10 kDa, >10 kDa) and enzyme treatment. Across all groups, peptides fractions >10 kDa contained the highest proportion of protein, especially in T10 (54.69 %) and C10 (51.71 %) fractions. Higher molecular weight peptides contributed more to protein content even if smaller number of peptides were generated. The peptides with lower molecular weight, such as T3–10(21.30 %), C3–10(22.91 %), C3 (25.38 %) and TC3 (25.22 %) exhibited lower protein content across all enzyme treatments. In general, although chymotrypsin achieved higher hydrolysis, it may generate a great number of smaller fragments (<3 kDa), while trypsin tended to produce larger peptide fragments (3–10 kDa or 10 kDa) that retain more total protein content.

### 3.2 Antimicrobial activity of peptide fractions

**Fig. 2:**
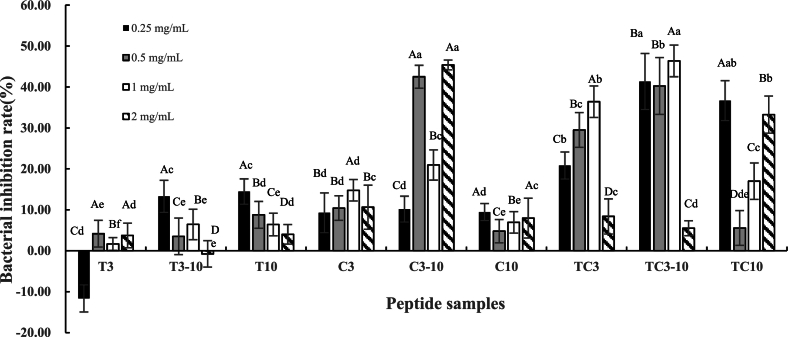
Bacterial inhibition rates of peptide fractions derived from *Chlamydomonas reinhardtii* protein hydrolysates obtained with trypsin (T), chymotrypsin (C), and trypsin–chymotrypsin combination (TC). Peptide fractions were classified by molecular weight as <3 kDa (3), 3–10 kDa (3−10), and > 10 kDa (10). Different uppercase letters (A–D) indicate statistically significant differences among concentrations (p < 0.05), while different lowercase letters indicate statistically significant differences among enzyme treatments and molecular weight fractions (p < 0.05). Negative antibacterial rates indicate ‘stimulation of bacterial growth’.

Fig. 2 shows the antibacterial activity of peptide fractions from different enzymatic treatments and molecular weight ranges. Low-molecular-weight fractions (<3 kDa) T3 and C3 exhibited weak inhibition (<10 % at 0.25–1 mg/mL), whereas TC3 displayed markedly higher activity (20.8–36.4 %). This suggests that dual-enzyme hydrolysis enhances antimicrobial potential by generating more diverse peptide sequences through complementary cleavage specificities. Trypsin preferentially cleaves after basic residues (Lys, Arg), while chymotrypsin targets bulky hydrophobic residues (Phe, Tyr, Trp, Leu). Interestingly, the T3 fraction even showed negative inhibition at 0.25 mg/mL, implying that some very small peptides may serve as nutrients at low concentration, thereby stimulating bacterial growth rather than suppressing it, which is consistent with reports of hormetic responses. For example, certain peptides at sub-μg/mL levels accelerate *Mycobacteria* enter into log phase and increase growth rate (Hilpert et al., 2023). Therefore, our negative inhibition at low concentration likely reflects that a subset of small peptides in fraction T3 serve as utilizable substrates by *Salmonella*, rather than exert direct antimicrobial action at that concentration.

The medium-molecular-weight fractions (3–10 kDa) exhibited the strongest antibacterial activity, though concentration–response patterns differed among treatments. TC3–10 showed a gradual, dose-dependent increase in inhibition (up to >45 % at 1 mg/mL, *p* < 0.05), whereas C3–10 displayed a sharper rise between 0.5 and 2 mg/mL, and T3–10 remained significantly less active. These results suggest that dual-enzyme hydrolysis were more effective in generating bioactive sequences within this size range, likely due to an optimal balance of amphipathic structure, diffusion capacity, and stability. In contrast, >10 kDa fractions displayed weaker and more variable effects. TC10 showed modest activity (5–37 %) without a clear dose-response, while T10 and C10 were largely inactive (<10 %) and inconsistent, possibly due to steric hindrance or aggregation.

Overall, the data revealed significant differences in antibacterial efficacy depending on both enzyme treatment and molecular weight. The dual-enzyme-treated fraction TC3–10 emerged as the most potent, exhibiting inhibition rates exceeding 45 % at relatively low concentrations (0.25–1 mg/mL, *p* < 0.05). This highlighted the synergistic role of combined proteolysis in generating bioactive peptides with enhanced antibacterial effects. Based on its superior performance, the TC3–10 fraction was selected for further investigation, including studies on antimicrobial mechanisms, peptide sequence identification, and in silico analysis to elucidate structure-activity relationships.

### 3.3 Cell membrane integrity

**Fig. 3:**
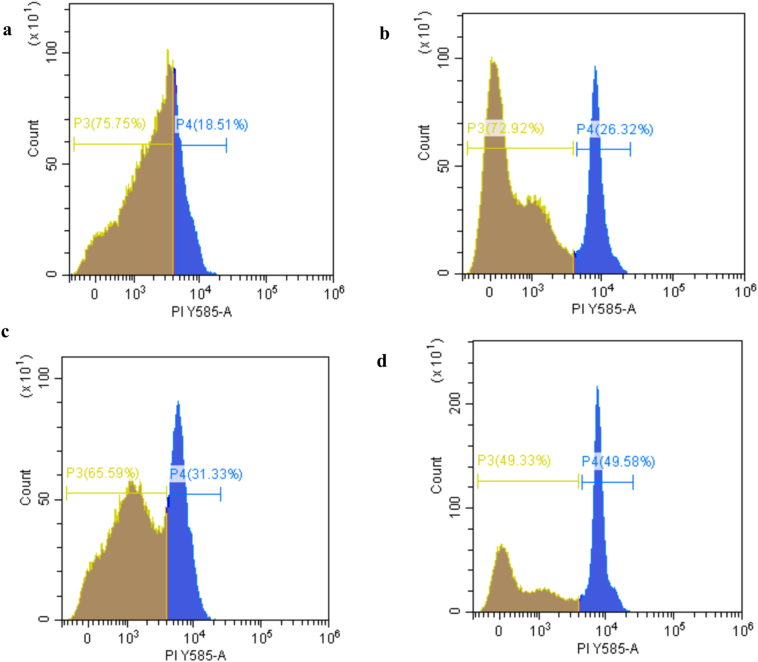
Flow cytometry analysis of peptide-induced effects on the membrane integrity of *Salmonella* cells. (a) Untreated control cells; (b–d) cells treated with TC3–10 peptide solutions at 15.625, 31.25, and 62.5 μg/mL, respectively. P3 (Proportion 3) represents the percentage of viable cells, while P4 (Proportion 4) represents the percentage of damaged cells stained with propidium iodide.

Propidium iodide (PI) staining is a widely used fluorescence-based method to evaluate bacterial membrane integrity, as it selectively penetrates cells with compromised membranes and binds to DNA, producing red fluorescence that indicates membrane damage or cell death. In this study, PI staining was used to assess membrane integrity of *Salmonella* after peptide treatment. Flow cytometry revealed a clear concentration-dependent increase in PI-positive cells, rising from 18.51 % in the control to 26.32 % and 31.33 % at 15.625 and 31.25 μg/mL, and reaching 49.58 % at 62.5 μg/mL (Fig. 3). These results indicate substantial membrane disruption at higher concentrations, consistent with the established mode of action of many AMPs.

**Fig. 4:**
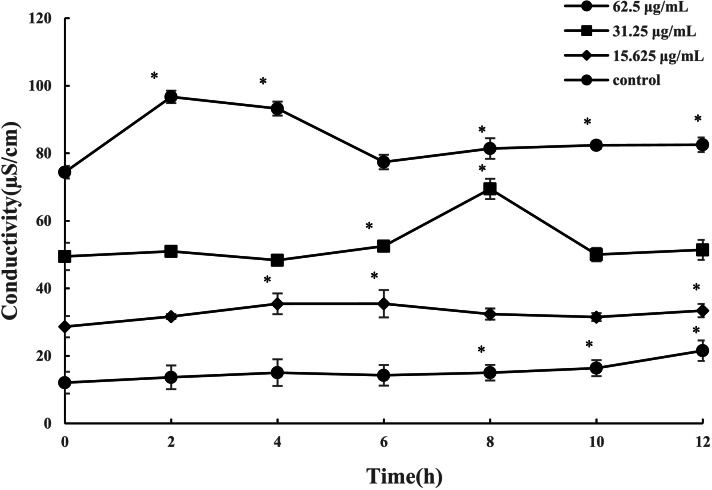
Effect of peptides on the conductivity of *Salmonella* suspensions over time. Data are presented as mean ± SD. An asterisk (*) indicates a statistically significant difference compared with 0 h of the corresponding sample (*p* < 0.05).

The integrity of the bacterial membrane is essential for maintaining ionic homeostasis between intracellular and extracellular environments. When the membrane is compromised, intracellular ions leak into the surrounding medium, leading to an increase in electrical conductivity. To further confirm membrane damage, electrical conductivity of bacterial suspensions was monitored over 12 h (Fig. 4). Peptides at 62.5 and 31.25 μg/mL induced sharp increases in conductivity within 2–8 h, reflecting rapid ion leakage across compromised membranes, followed by partial decreases. The subsequent decrease in conductivity may be explained by the interaction between extracellular ions and peptides(Zhang et al., 2017). By contrast, only minor changes were detected at 15.625 μg/mL and in controls, suggesting insufficient disruption at low concentration. Together, these results demonstrate that the peptides act primarily through dose-dependent membrane permeabilization leading to ionic imbalance and cell death.

### 3.4 Leakage of intracellular substances

**Fig. 5:**
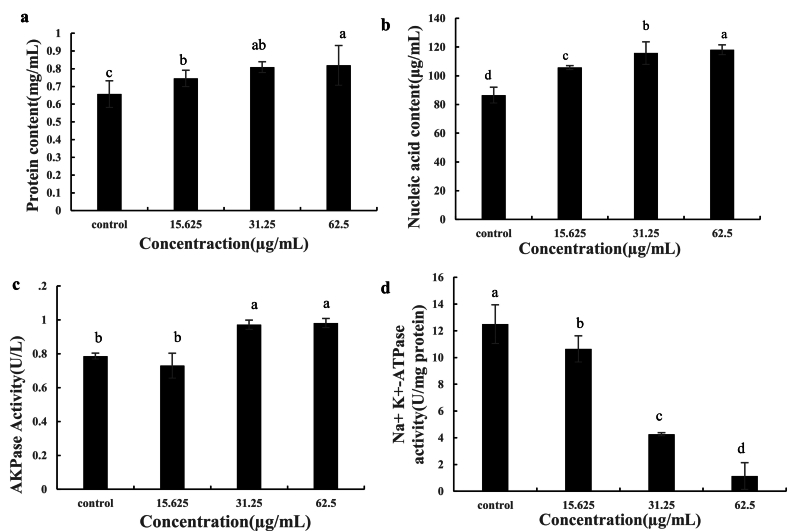
Effect of peptides on (a) protein content, (b) nucleic acid content, (c) AKPase activity, and (d) Na^+^/K^+^-ATPase activity of *Salmonella* spp. cells. Different superscript letters indicate statistically significant differences among treatments (*p* < 0.05).

Fig. 5(a) and 5(b) illustrate the concentration-dependent leakage of intracellular protein and nucleic acids from *Salmonella*. As shown in Fig. 5(a), the protein leakage increased significantly with rising peptide concentration, reaching a maximum at 62.5 μg/mL(0.83 mg/mL), significantly higher than the control group (0.69 mg/mL, *p* < 0.05). A similar trend is observed in Fig. 5(b), where nucleic acid leakage peaked at 62.5 μg/mL. These results suggested that the algal-derived peptides compromised the bacterial cell membrane integrity, leading to leakage of cytoplasmic components.

### 3.5 Cell metabolism enzymes

Alkaline phosphatase (AKPase) is a widely distributed intracellular enzyme that hydrolyzes phosphate monoesters, releasing inorganic phosphate and the corresponding alcohols. It plays a key role in nucleic acid metabolism and in the phosphrylation-dephosphorylation of proteins. As shown in Fig. 5(c), the control group and the 15.625 μg/mL treatment showed no significant difference (*p* > 0.05), both maintaining relatively low AKPase activity levels, and no significant difference was observed between the 31.25 μg/mL and 62.5 μg/mL treatments (p > 0.05), indicating that the stimulatory effect of TC3–10 on AKPase activitywas minimal.

Na^+^-K^+^ ATPase, is an important enzyme located on the cell membrane. It is responsible for maintaining the concentration gradients of sodium ions (Na^+^) and potassium ions (K^+^), which are crucial for the normal physiological functions of the cell. As illustrated in Fig. 5(d), treatment with *C. reinhardtii*-derived peptides (TC3–10) resulted in a concentration-dependent decrease in Na^+^/K^+^-ATPase activity in *Salmonella.* The control group exhibited the highest enzyme activity (12.5 U/mg protein), whereas peptide treatments progressively reduced activity 10.6, 4.3, and 1.1 U/mg protein at 15.625, 31.25, and 62.5 μg/mL, respectively (p < 0.05). This decline indicates that the peptides impaired ion transport homeostasis by disrupting membrane-bound ATPase function, thereby compromising electrochemical gradient maintenance.

### 3.6 In silico analysis of peptide sequences

As shown in Table 1, in silico functional screening was performed on 26 peptides isolated from TC3–10. All peptides achieved PeptideRanker scores above 0.7, ranging from 0.71 (HAYPGPGP) to 0.97 (KFPGF, WAMP), indicating high predicted bioactivity. These scores suggested strong potential for biological activities, such as antimicrobial or immunomodulatory effects, consistent with the PeptideRanker model (Mooney et al., 2012).

The peptides displayed GRAVY values from −2.72 to +0.78, covering a wide spectrum of hydrophilicity to hydrophobicity. Strongly negative GRAVY values (e.g., NPDPRP, SPDPKRP) indicate high hydrophilicity and enrichment in polar amino acids, features that enhance solubility and facilitate interactions with aqueous environments or intracellular targets. Conversely, peptides with moderately positive GRAVY values (e.g., LSRPFL, GSPMAL) are more hydrophobic, favoring membrane interaction, an essential property of antimicrobial peptides (AMPs).

The Boman index, which estimates peptide–protein binding potential, varied significantly from −1.52 to +5.04 kcal/mol. Several peptides, such as NPDPRP, DQRLF, and FPDEPR exhibited high Boman scores (>4.0), suggesting a strong propensity to interact with intracellular proteins or enzymes. Such interactions point to possible roles beyond membrane disruption, including immune signaling or enzyme inhibition (Torrent et al., 2011). The peptide sequence lengths ranged from 4 to 8 amino acids, consistent with bioactive short peptide motifs, known for enhanced stability, good cell permeability, and low immunogenicity (Nakamura et al., 2011). Importantly, none of the peptides were flagged as toxic, reinforcing their potential for applications in food, pharmaceutical, or cosmetic formulations. This observation aligns with previous findings on algal-derived peptides, which are generally biocompatible and non-hemolytic as demonstrated in studies on *Chlorella pyrenoidosa* hydrolysate (Yaghoubzadeh & Safari, 2025).

Further, ITScorePeP, a knowledge-based scoring function optimized for peptide–protein interactions, was applied to evaluate peptide binding affinity(Xu et al., 2018). Docking scores ranged from −135.0 to −60.3 for 5v2w and from −133.6 to −45.8 for 5ztj, suggesting consistently strong but variable binding potential. Among the tested peptides, EWRPF (Peptide 5) showed one of the strongest binding affinities toward both proteins, with scores of −132.4 (5v2w) and − 133.6 (5ztj), highlighting its potential to interfere with multiple stages of bacterial DNA supercoiling or replication, and toxic expression regulation. Similarly, DQRLF (Peptide 17) achieved the lowest binding energy to 5v2w (−135.0) and maintained strong binding with 5ztj (−103.8), suggesting potent multi-target inhibitory activity. Based on these results, peptide EWRPF was selected for subsequent binding site analysis.

### 3.7 Molecular docking

The molecular docking analysis between peptide EWRPF and two target proteins, 5v2w and 5ztj, revealed multiple potential binding sites and stable interactions, indicating a strong bioactivity potential. As shown in panels a and b, four distinct binding pockets were identified on each protein, suggesting multiple possible modes of peptide accommodation.

**Fig. 6:**
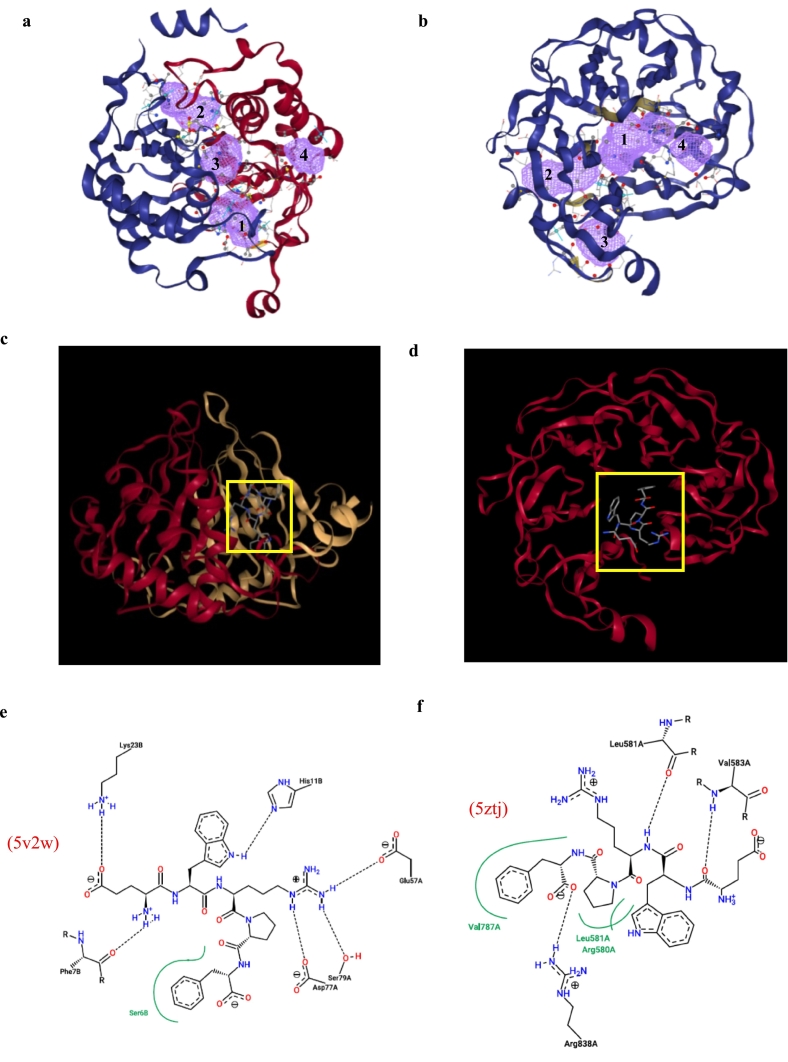
Molecular docking results of peptide EWRPF with proteins 5v2w and 5ztj. Panels a and b show predicted binding pockets, panels c and d illustrate the 3D binding conformations, and panels e and f depict the corresponding 2D interaction diagrams. Proteins are represented in cartoon format, while the peptide is shown in licorice representation. In panels a and b, the purple regions with numbered labels indicate distinct molecular docking pockets. (For interpretation of the references to colour in this figure legend, the reader is referred to the web version of this article.)

Table 3 summarized the structural properties of the predicted binding pockets of LuxS (5v2w) and the GyraseA C-terminal domain (5zjt). The structural parameters indicate four potential ligand-binding sites in each enzyme, with pocket 1 showing the highest likelihood of interaction, followed by pockets 2–4 in decreasing order. Pocket 1 of protein 5v2w (Table 3) exhibited the highest binding likelihood, with a volume of 175.10 Å^3^ and a surface area of 315.09 Å^2^, yielding a surface-to-volume ratio of 1.80, suggested a moderately enclosed cavity, providing both accessibility and specificity by offering sufficient interaction surface while partially shielding the ligand from solvent exposure (Weisel et al., 2007). The pocket contained 84 protein heavy atoms, creating a dense interaction environment that facilitates multiple non-covalent contacts. With 17 hydrogen bond acceptors and 11 donors the site showed strong potential for polar interactions. The hydrophobicity index of 0.58 further indicated a moderately hydrophobic environment, favoring amphiphilic peptides whose hydrophobic residues can interact with non-polar regions while polar or charged residues engaged in hydrogen bonding (Sable & Jois, 2015). A comparison of Fig. 6**a** and **c** suggested that the peptide is likely to interact with the enzyme in the vicinity of pockets 1 and 3.

By comparison, pocket 1 of 5ztj displayed distinct structural and chemical properties. It was significantly larger (324.61 Å^3^) and more spacious (528.52 Å^2^), along with a greater depth (15.45 Å), suggesting a deeply embedded binding site. While the number of hydrogen bond acceptors (17) was the same, 5ztj contained slightly more donors (13 vs 11), and exhibited a lower hydrophobicity index (0.53 vs. 0.58), pointing to a more balanced amphipathic environment (Liang et al., 1998). A comparison of Fig. 6**b** and **d** indicated that the peptide binds in the pocket 1. These characteristics suggested that 5ztj is more likely to facilitate flexible, deep-seated peptide binding, where s 5v2w may promote more specific, structurally constrained interactions, potentially influenced by metal coordination. Therefore, the same peptide could exhibit distinct binding modes and affinities depending on the protein target.

Panels c and d show the 3D binding conformations, where EWRPF is stably accommodated within the binding pockets (peptide in licorice, proteins in cartoon format). The corresponding 2D interaction maps (Panels e and f) highlight distinct binding modes. In 5v2w, EWRPF formed hydrogen bonds with His118, Gln87A, Ser77A, and Asn97A, along with electrostatic and π-stacking interactions with Lys238 and Phe176, indicating a compact, metal-stabilized binding mode. In contrast, binding to 5ztj involved a broader hydrogen-bonding network with residues such as Leu518A, Arg380A, and Val787A. The absence of a metal ion in 5ztj was offset by enhanced polar interactions, yielding a more flexible conformation. These differences suggest that EWRPF adapts to distinct molecular environments, which may underlie its broad-spectrum antimicrobial potential.

Taken together, these multi-pocket docking observed here suggested that EWRPF, derived from *C. reinhardtii* hydrolysates, may exert antimicrobial effects not only by disrupting membrane integrity but also by interfering with intracellular targets such as enzymes or nucleic acid-associated proteins.

## 4 Discussion

*Salmonella* is a Gram-negative pathogen whose outer membrane, enriched with lipopolysaccharides (LPS), provides strong intrinsic resistance to conventional antimicrobials (Murray et al., 2022). While many studies have investigated longer or single-mechanism antimicrobial peptides, evidence on short algal-derived peptides remains scarce. Our findings demonstrate, for the first time, that peptide fractions from *C. reinhardtii* hydrolysates exhibit potent activity against *Salmonella*, with dual in vitro and in silico analyses confirming their ability to compromise membrane integrity and interact with intracellular targets. Notably, the TC3–10 fraction emerged as the most effective, highlighting both the value of dual-enzyme hydrolysis and the underexplored potential of mid-molecular-weight algal peptides as novel antimicrobial agents.

The enzymatic hydrolysis of *Chlamydomonas reinhardtii* proteins revealed that chymotrypsin achieved the highest degree of hydrolysis (DH) at 72.51 %, significantly surpassing trypsin (67.59 %) and the trypsin–chymotrypsin (TC) combination (64.89 %). This finding is consistent with previous studies indicating that chymotrypsin preferentially cleaves peptide bonds adjacent to hydrophobic residues such as phenylalanine, tyrosine, and tryptophan, which are abundant in algal proteins (Amiri et al., 2025; Bechinger & Gorr, 2017). The slightly lower DH observed in the TC system may be attributed to steric hindrance or competition between the two enzymes at overlapping cleavage sites, leading to suboptimal hydrolysis efficiency (Sun et al., 2016). Despite the higher DH achieved by chymotrypsin, the TC hydrolysates exhibited a more diverse peptide profile, particularly in the 3–10 kDa range, correlating with enhanced antimicrobial activity. This suggests that peptide diversity and sequence specificity may outweigh hydrolysis efficiency alone in determining bioactivity. Similar observations have been reported in hydrolysates derived from *Chlorella vulgaris* and *Spirulina maxima*, where specific peptide sequences contributed to superior bioactive properties (Sun et al., 2016; Yaghoubzadeh & Safari, 2025).

Protein content analysis indicated that peptides larger than 10 kDa retained the highest protein levels, especially in trypsin hydrolysates, likely due to incomplete cleavage. However, these larger peptides exhibited weak antimicrobial activity. In contrast, the TC 3–10 kDa fraction, despite its relatively lower protein yield, demonstrated superior antimicrobial efficacy, highlighting the importance of peptide sequence and physicochemical characteristics over total peptide mass in determining bioactivity.

Interestingly, antimicrobial activity did not increase linearly with concentration, At higher concentrations (e.g. > 2 mg/mL), inhibition plateaued or fluctuated, especially in the C3–10 and TC3–10fractions. This non-dose-dependent behavior has been reported previously and may result from peptide aggregation or self-assembly into micelle-like structures that hinder their diffusion (Shai, 2002). Additionally, peptide–peptide interactions, including hydrogen bonding or hydrophobic clustering, may mask critical cationic or amphiphilic residues, reducing membrane binding efficiency (Shai, 2002).

Mechanistic studies confirmed that the TC3–10 peptide fraction exerts antimicrobial activity mainly through membrane disruption. PI staining and conductivity assays showed concentration-dependent increases in membrane permeability, with nearly 50 % of *Salmonella* cells PI-positive at 62.5 μg/mL and accompanied by elevated extracellular conductivity. Consistent with a lytic killing mechanism, biochemical assays revealed dose-dependent leakage of proteins and nucleic acids. These results align with reports of algal-derived AMPs such as ALFPm3 (Li et al., 2022), which destabilize bacterial membranes, while differing from non-lytic peptides like Buforin II (Park et al., 1998). Furthermore, Na^+^/K^+^-ATPase activity was inhibited after peptide treatment, resembling the mechanism of *Tetraselmis suecica* peptides that disrupt ionic balance (Guzmán et al., 2019), whereas alkaline phosphatase showed no consistent changes. Overall, these findings indicate a multi-level antimicrobial mechanism.

In current studies, AMPs display a wide structural diversity, ranging from short Trp/Arg-rich motifs to amphipathic α-helices and proline-rich linear chains, and these features strongly determine their mechanisms of action. Classical membrane-active AMPs, such as magainins and many host defense peptides, are typically cationic, amphipathic, and sufficiently long (12–40 residues) to form stable α-helices or β-sheets that disrupt lipid bilayers (Bechinger & Gorr, 2017). By contrast, intracellular-targeting peptides often contain high levels of proline and/or arginine and can penetrate cells without extensive lysis. The peptide candidates identified in Table 2 are unusually short (5–8 residues) but contain functionally critical amino acids, including tryptophan (W), phenylalanine (F), arginine (R), and proline (P). Short Trp-Arg motifs are known to retain antimicrobial activity despite minimal length (Scocchi et al., 2011), consistent with the properties of EWRPF. Structurally, EWRPF resembles short Trp-Arg membrane-active peptides while its proline content may also favor cell penetration. Docking simulations further revealed strong binding of EWRPF to LuxS and Gyrase A, indicating potential interference with both quorum sensing and DNA supercoiling. Collectively, these results suggest that EWRPF is multifunctional, combining membrane disruption with intracellular targeting, whereas many AMPs act predominantly through a single mechanism. Such multifunctionality may be therapeutically advantageous by reducing the likelihood of resistance development.

**Table 2:** In silico characterization of TC3–10–derived peptides with predicted bioactivity scores, physicochemical properties, toxicity assessment, and docking affinities (5v2w, 5ztj).

	Peptide	ALC(%)	Length	Molecular	Charge	GRAVY	Boman (kcal/mol)	Peptide ranker score	Tocixity	ITScorePeP	ITScorePeP (5ztj)
	sequence			weight						(5v2w)	
1	KFGPF	95	5	503.66	1	−0.06	−0.27	0.97	–	−117.7	−111.1
2	WAMP	93	4	540.62	0	0.3	–	0.97	–	−101.2	−84.1
3	NNFF	90.6	4	696.93	0	−0.35	–	0.95	–	−101.1	−95.4
4	WPPGLK	90.2	6	733.88	1	−0.76	−0.43	0.95	–	−111.8	−111.3
5	EWRPF	97.9	5	734.87	0	−1.54	3.28	0.94	–	−132.4	−133.6
6	FPDPYP	95.3	6	606.74	−1	−1.13	0.98	0.93	–	−109.3	−107.6
7	HAPWP	90.1	5	820.97	0	−1.1	0.1	0.92	–	−127.9	−107.5
8	SEWRFP	96.4	6	380.48	0	−1.42	3.3	0.9	–	−130.6	−131.7
9	APPP	91.2	4	731.97	0	−0.75	–	0.9	–	−60.3	−45.8
10	LSRPFL	90	6	647.85	1	0.58	0.91	0.88	–	−117.8	−110
11	SFPGLK	93.6	6	453.59	1	−0.02	0.01	0.84	–	−108.6	−95.4
12	QPPL	93.9	4	574.77	0	−0.08	–	0.82	–	−78.9	−58.6
13	GSPMAL	90.6	6	694.81	0	0.78	−1.1	0.82	–	−94.8	−76.7
14	NPDPRP	96	6	887.01	0	−2.72	5.04	0.77	–	−85.4	−81.6
15	DGAAPDWR	97.9	8	653.87	−1	−1.35	3.18	0.77	–	−127.6	−126.6
16	AAVCPPP	97.9	7	677.82	0	0.79	−1.27	0.77	–	−76.3	−70.7
17	DQRLF	90.5	5	795.97	0	−0.8	4.25	0.77	–	−135	−103.8
18	SPDPKRP	91.1	7	471.63	1	−2.5	4.65	0.76	–	−110.8	−89.8
19	CVGPP	91.2	5	606.74	0	0.62	−1.25	0.75	–	−72	−52.6
20	DFLGR	93.3	5	715.84	0	−0.36	2.96	0.74	–	−126.1	−100
21	GEGWGGGP	90	8	729.95	−1	−1	−0.02	0.74	–	−119.3	−104.3
22	FPEPKL	92	6	706.87	0	−0.67	0.74	0.73	–	−116.5	103
23	DSYPLL	91.4	6	759.88	−1	0.07	0.4	0.73	–	−115.4	107.5
24	DPFEPR	98.4	6	618.79	−1	−1.98	4.57	0.72	–	−116.3	106.5
25	LPDFK	94.9	5	794.97	0	−0.48	1.27	0.72	–	−104.9	−94.3
26	HAYPGPGP	93.6	8	795.37	0	−1.04	0.13	0.71	–	−120.7	−108.7

**Table 3:** Pocket properties in molecular docking of 5v2w and 5ztj.

	NO.	Depth	Surface	Volume	Surface-volume ratio	Protein heavy atoms	Donors	Acce-ptors	Hydro-phobic-ity
		(Å)	(Å^2^)	(Å^3^)					
5v2w	1	9.36	315.09	175.1	1.8	84	11	17	0.58
	2	9.26	292.76	160.26	1.83	61	6	13	0.59
	3	7.07	75.85	102.4	0.74	63	2	4	0.7
	4	6.3	198.14	73.73	2.69	40	0	4	0.78
5ztj	1	15.45	528.52	324.61	1.63	106	13	17	0.53
	2	11.45	358.1	141.31	2.53	53	3	9	0.58
	3	7.02	217.35	98.3	2.21	42	5	8	0.57
	4	5.12	253.48	52.22	4.85	33	4	5	0.55

In this study, although molecular docking provided mechanistic insights, MIC and MBC assays were not performed to confirm the antimicrobial potency of the identified peptides, mainly due to the high cost of peptide synthesis and limited laboratory facilities. In addition, the extraction and hydrolysis conditions were adopted from established literature rather than optimized experimentally; while these parameters ensured reproducibility, further optimization may enhance peptide yield and activity. Another limitation is the observation that the low-molecular-weight T3 fraction (<3 kDa) showed “negative inhibition” at 0.25 mg/mL. We speculated that some small peptides may act as nutrients at low concentrations and stimulate bacterial growth, a hypothesis supported by previous reports (Hilpert et al., 2023). Future studies should therefore include systematic MIC/MBC testing, optimization of extraction/hydrolysis parameters, and targeted experiments such as growth curve analysis or metabolomic profiling to confirm the nutritional utilization of small peptides.

## 5 Conclusion

In summary, this study provides the first systematic evidence that protein hydrolysates from *Chlamydomonas reinhardtii* contain bioactive peptides with significant antimicrobial activity against *Salmonella*. The most active fraction (TC3–10) displayed both membrane-disrupting and intracellular inhibitory effects, while molecular docking identified EWRPF as a high-affinity peptide candidate targeting bacterial proteins. The integration of biochemical assays with computational analyses represents a key innovation, enabling efficient identification of promising antimicrobial peptides. These findings highlight *C. reinhardtii* as a novel source of sustainable bio-preservatives and provide a mechanistic foundation for their application. Future studies could focus on validating these peptides through in vitro models, optimizing production strategies, and exploring formulation approaches to enable their practical application in food systems and beyond.

## CRediT authorship contribution statement

**Keying Su:** Writing – original draft, Methodology, Investigation, Formal analysis, Data curation. **Lecheng Wu:** Investigation, Data curation. **Yating Lin:** Software, Investigation, Data curation. **Qian Li:** Software, Project administration. **Hua Liu:** Investigation, Data curation. **Xuewu Zhang:** Writing – review & editing, Supervision, Methodology, Funding acquisition, Conceptualization. **Lai-Hoong Cheng:** Writing – review & editing, Supervision, Conceptualization.

## Declaration of generative AI and AI-assisted technologies in the writing process

During the preparation of this work the author(s) used ChatGPT to improve language. After using this tool, the author(s) reviewed and edited the content as needed and took full responsibility for the content of the published article.

## Funding sources

This work was supported by the Young Innovative Talents Project of General Colleges and Universities, Department of Education of Guangdong Province, China [grant numbers: 2025KQNCX113], Characteristic Innovation Project (Natural Science) for Ordinary Universities in Guangdong Province, China [grant numbers: 2024KTSCX151] and Project for Improving the Scientific Research Ability of Key Disciplines under Construction in Guangdong Province, China [grant numbers:2024ZDJS090].

## Declaration of competing interest

The authors declare that they have no known competing financial interests or personal relationships that could have appeared to influence the work reported in this paper.

## Data Availability

The datasets generated and analyzed during the current study are not publicly available due to institutional and laboratory regulations. However, the data can be made available from the corresponding author upon reasonable request.
